# Comparative analyses of the in vivo induction and transmission of α-synuclein pathology in transgenic mice by MSA brain lysate and recombinant α-synuclein fibrils

**DOI:** 10.1186/s40478-019-0733-3

**Published:** 2019-05-20

**Authors:** Jess-Karan S. Dhillon, Jorge A. Trejo-Lopez, Cara Riffe, Yona Levites, Amanda N. Sacino, David R. Borchelt, Anthony Y. Yachnis, Benoit I. Giasson

**Affiliations:** 1 Department of Neuroscience, University of Florida, Gainesville, FL 32610 USA; 2 Center for Translational Research in Neurodegenerative Disease, University of Florida, Gainesville, FL 32610 USA; 3 Department of Pathology, University of Florida, Gainesville, FL 32610 USA; 4 McKnight Brain Institute, University of Florida, Gainesville, FL 32610 USA

**Keywords:** α-Synuclein, Multiple system atrophy, Prion, Seeding, Transgenic

## Abstract

α-synuclein (αS) is the major component of several types of brain pathological inclusions that define neurodegenerative diseases termed synucleinopathies. Central nervous system (CNS) inoculation studies using either in vitro polymerized αS fibrils or in vivo derived lysates containing αS aggregates to induce the progressive spread of αS inclusion pathology in animal disease models have supported the notion that αS mediated progressive neurodegeneration can occur by a prion-like mechanism. We have previously shown that neonatal brain inoculation with preformed αS fibrils in hemizygous M20^+/−^ transgenic mice expressing wild type human αS and to a lesser extent in non-transgenic mice can result in a concentration-dependent progressive induction of CNS αS pathology. Recent studies using brain lysates from patients with multiple system atrophy (MSA), characterized by αS inclusion pathology in oligodendrocytes, indicate that these may be uniquely potent at inducing αS pathology with prion-like strain specificity. We demonstrate here that brain lysates from MSA patients, but not control individuals, can induce αS pathology following neonatal brain inoculation in transgenic mice expressing A53T human αS (M83 line), but not in transgenic expressing wild type human αS (M20 line) or non-transgenic mice within the timeframe of the study design. Further, we show that neuroanatomical and immunohistochemical properties of the pathology induced by MSA brain lysates is very similar to what is produced by the neonatal brain injection of preformed human αS fibrils in hemizygous M83^+/−^ transgenic mice. Collectively, these findings reinforce the idea that the intrinsic traits of the M83 mouse model dominates over any putative prion-like strain properties of MSA αS seeds that can induce pathology.

**Electronic supplementary material:**

The online version of this article (10.1186/s40478-019-0733-3) contains supplementary material, which is available to authorized users.

## Introduction

Amid the group of neurodegenerative diseases known as α-synucleinopathies, characterized by the formation of aberrant α-synuclein (αS) pathological inclusions, multiple system atrophy (MSA) represents a unique entity. Unlike Parkinson’s disease (PD) and dementia with Lewy bodies (DLB) where αS inclusions predominantly occur in neuronal populations, the signature αS inclusion in MSA is observed in glial cells, accumulating in the form of the argyrophilic glial cytoplasmic inclusions (GCIs) [44, 47]. Additionally, MSA presents as a markedly aggressive clinical disease, with a median time of survival from diagnosis of approximately 9.5 years [52]. GCIs can be identified across multiple regions of the neuraxis, with neuronal degeneration and loss correlating with GCI burden [20, 48]. Expression of αS exclusively in glia produces GCIs as well as neuronal injury and loss, suggesting that GCI αS might in fact serve as the root pathological insult of neurodegeneration in MSA [58]. However, it is still unclear how a protein that is predominantly expressed in neurons [14, 21, 22, 53] preferentially aggregates within oligodendrocytes in MSA.

Cell culture and experimental animal models have provided compelling evidence to suggest that the aberrant aggregation of αS is capable of spreading through the central nervous system via a “prion-like” conformational templating mechanism. Pathologic forms αS are thought to seed and induce the misfolding of native normal αS, propagating disease from cell to cell and throughout the nervous system via neuroanatomical pathways [6, 18, 19, 46]. Similar to authentic prion disease driven by the prion protein, PrP, the concept of different strains of aggregated αS is emerging, in which distinct forms of misfolded αS, exhibiting different structural and biophysical properties, can produce distinct disease phenotypes [5, 27]. This αS strain model represents a particularly compelling concept when applied to MSA, as it may provide an explanation for the idiosyncratic role of GCIs in the pathophysiology of this disease. Evidence for distinct strains of misfolded αS in MSA includes the observation that αS fibrils within GCIs are wider and more tubular than the typical αS neuronal inclusions found in PD and DLB [17, 26, 51]. Additionally, recent experimental modeling studies implicate the intracellular environmental milieu of the oligodendrocytes in producing a GCI-specific strain of αS [28]. It was suggested that passaging of pre-formed human αS fibrils through oligodendrocytes produces an αS strain with significantly more potent seeding capabilities than those produced by passage through neurons [28]. Moreover, previous investigations utilizing mouse models of seeding pathology through intracerebral injections with brain tissue samples derived from human patients with either PD or MSA have shown MSA to be uniquely capable of inducing pathology [30].

To further investigate the unique seeding ability of MSA derived αS and the possibility of specific conformation templating strain properties, we utilized neonatal mouse brain to screen for induction of αS pathology [37]. The use of P0 mouse pups allows for rapid completion of experimental cohorts and building on previous studies in which neonatal mice were injected intracerebroventricularly with recombinant adeno-associated virus [8], which allowed for widespread transduction of the neonatal mouse brain, we conducted a comparison of pathology induced by MSA brain lysates in hemizygous M83^+/−^ mice that express A53T human αS, hemizygous M20^+/−^ mice that express human wild type (WT) αS, and non-transgenic (nTg) mice with the same genetic background. In this experimental paradigm, M83^+/−^ mice were significantly more susceptible to induced αS pathology by MSA seeds, than in M20^+/−^ or nTg mice. We further compared the histochemical properties and neuroanatomical distribution of αS pathology induced by MSA-derived αS seeds to the pathology induced by preformed αS fibrils. Our findings demonstrate that the intrinsic properties of the A53T αS in the M83 mouse model dominate over any strains features harbored by misfolded αS in MSA brains.

## Methods

### αS recombinant protein purification and fibril formation

The pRK172 bacterial expression vectors containing cDNA encoding WT or A53T full-length human αS were generated as previously described [16, 34, 35]. Plasmids were transformed into BL21 (DE3)/RIL *Escherichia coli* (*E. coli*; Agilent Technologies) and recombinant αS was purified from *E. coli* by size exclusion chromatography and subsequent anion exchange as previously described [16]. Protein concentrations were determined by bicinchoninic acid assay using bovine serum albumin as the protein standard. Recombinant αS proteins (5 mg/ml in sterile phosphate buffered saline; PBS) were incubated at 37 °C with constant shaking at 1050 rpm (Thermomixer R, Eppendorf) for > 48 h. Fibril formation was monitored by K114 [(*trans*, *trans*)-1-bromo-2,5-bis-(4-hydroxy)styrylbenzene] fluorometry as previously described [11]. To prepare fibrils for injection, fibrils were diluted to 2 mg/ml in sterile PBS and sonicated in a water bath for 2 h. Sonicated fibrils were then aliquoted, stored at − 80 °C and thawed when required. Each experiment in this study was performed using fibrils from the same preparation, limiting batch to batch variation.

### Antibodies

94-3A10, 71E10, 9C10, and 33A-3F3 monoclonal anti-αS specific mouse antibodies were previously generated and described in Dhillon et al. 2017 [12]. 9C10 preferentially reacts with aggregated αS. αS phospho-Ser129 (pSer129) antibody 81A was previously characterized [31, 50] and EP1536Y was obtained from Abcam (Cambridge, MA). Rabbit antibody to p62 (SQSTM1; Proteintech, Chicago, IL) is a general marker of inclusion pathology. Anti-glial fibrillary acid protein (GFAP) (Dako, Agilent, Carpentaria, CA), and CD11B (Abcam, Cambridge, MA) are reactive for astrocytes or microglia, respectively. Rabbit antibody to tubulin polymerization-promoting protein/p25α (Novus Biologicals, Centennial, CO) was obtained from Fisher Scientific.

### Human sample preparation

Cerebellar white matter from two control, elderly individuals without clinical evidence of a neurological illness, and two MSA individuals were obtained from the University of Florida Neuromedicine Brain Bank (Table 1). Post-mortem pathological diagnoses were made according to neuropathological criteria proposed by the Neuropathology Working Group on MSA [44]. These patients were chosen based on prior neuropathological study showing extensive pathology in the white matter. Cerebellar white matter homogenates were prepared as described in Eisele et al., 2009 [13]. Briefly, tissue was homogenized at 10% (w/v) in sterile PBS, vortexed, sonicated 3 × 5 sec and centrifuged at 3000×g for 5 min. Supernatant was aliquoted and immediately frozen as 10% extract.

**Table 1:** Demographics of human cases used to generate experimental lysate

	Age at onset	Age at death	Pathology diagnosis	Braak stage	Thal phase	CERAD score
Control 1	N/A	82	Cerebrovascular arteriolosclerosis	I/II	2	C1
Control 2	N/A	52	No neuropathology diagnosis	I/II	2	C1
MSA 1	58	67	MSA-C/OPCA	–	0	C0
MSA 2	68	71	MSA-P/SND	–	2	C1

*MSA-C* multiple system atrophy-cerebellar, *OPCA* olivo-ponto-cerebellar atrophy, *MSA-P* multiple system atrophy-parkinsonism, *SND* striatonigral degeneration

### Mouse lines

All procedures were performed according to the National Institute of Health Guide for the Care and Use of Experimental Animals and were approved by the University of Florida Institutional Animal Care and Use Committee. M20 and M83 transgenic mice on the C57BL/C3H background were previously described [15]. The M20 line is transgenic for WT human αS and the M83 line is transgenic for human αS with the pathogenic A53T mutation. Both αS transgenic mouse lines were generated with similar constructs with expression driven by the mouse prion protein promoter resulting in widespread CNS expression and similar expression, although expressing in the M20 line is slightly higher (Additional file 1: Figure S1) [4, 15, 38]. nTg mice on the same C57BL/C3H background were also used.

### Mouse experimental procedures

M83 mice were maintained as homozygous mice and were mated with nTg C3H/BL6 mice to generate neonatal M83^+/−^ for injections. M20 mice were maintained as hemizygous mice and were mated with nTg C3H/BL6 mice to generate both neonatal M20^+/−^ and littermate nTg control mice that were used for neonatal injections and genotyped thereafter. Neonatal M83^+/−^, M20^+/−^, and nTg mice were injected with 2 μl of brain homogenate or PBS control into both hemispheres using a 10 ml Hamilton syringe with a 30 g needle on day P0 as previously described [8, 37]. Mice were aged 5 month or until they developed hindlimb paralysis, whichever came first. Harvesting, fixation, and processing were conducted as previously described [36]. Similarly, some neonatal M83^+/−^ mice were bilaterally injected with either 2 μl of WT or A53T human αS fibrils (5 mg/ml) and aged for 4 months for comparison. Briefly, mice were euthanized by CO_2_, followed by cardiac perfusion of PBS/heparin. Brain and spinal cord were harvested and fixed in 70% ethanol/150 mM NaCl. Tissues were dehydrated and embedded in paraffin, then cut into 5 μm sections using a microtome. The number of animals analyzed, and their genotypes, are summarized in Table 2.

**Table 2:** Summary of neonatal P0 mouse brain inoculation studies

Type of Inoculums	Mouse Line Injected		
	M83^+/−^	M20^+/−^	nTg
MSA Case 1	6/12	0/5	0/4
MSA Case 2	3/12	0/8	0/6
Control Case 1	0/8	0/4	0/4
Control Case 2	0/8	0/16	0/11
PBS	0/20	0/5	0/6
WT αS Fibrils	5/5		
A53T αS Fibrils	8/8		

Number of mice with induced αS inclusion pathology per total number of mice injected for each cohort. Mouse lines: M83^+/−^ and M20^+/−^ transgenic mice and nTg mice. Inoculums: white matter cerebellum extracts from MSA or control individuals, PBS or in vitro aggregated recombinant WT or A53T human αS proteins

### Immunoblotting analyses

Protein samples were resolved by electrophoresis on 15% polyacrylamide gels, then electrophoretically transferred to 0.2 μm pore size nitrocellulose membranes in carbonate transfer buffer (10 mM NaHCO3, 3 mM Na2CO3, pH 9.9, 20% methanol). Membranes were blocked with 5% milk in Tris-buffered saline (TBS) for 1 h, then incubated overnight at 4 °C with primary antibodies diluted in 5% milk/TBS. Following washing with TBS, blots were incubated with HRP conjugated goat anti-mouse secondary antibodies (Jackson Immuno Research Labs, West Grove, PA) diluted in 5% milk/TBS for 1 h. Following washing with TBS, protein bands were visualized using Western Lightning-Plus ECL reagents (PerkinElmer, Waltham, MA) and images were captured using the GeneGnome XRQ system and GeneTools software (Syngene, Frederick, MD).

### Histological analyses

Paraffin embedded, formalin fixed human brain tissue was obtained through the University of Florida Neuromedicine Human Brain Tissue Bank. For both human and mouse tissues, sequential tissue sections were deparaffinized with xylenes, and sequentially rehydrated with graded ethanol solutions (100–70%). Antigen retrieval was performed by incubating sections in 0.05% Tween-20 in a steam bath for 60 min. For human tissue stained with αS antibodies an additional antigen retrieval step of 70% formic acid for 10 min was performed. Endogenous peroxidase activity was quenched with 1.5% hydrogen peroxide/0.005% Triton-X-100/PBS for 20 min. Sections were blocked with 2% FBS/0.1 M Tris, pH 7.6 then incubated with primary antibody overnight at 4 °C. Following washing with 0.1 M Tris, pH 7.6, sections were incubated with biotinylated horse anti-mouse or biotinylated horse anti-rabbit antibodies (Vector Laboratories) diluted in 2% FBS/0.1 M Tris pH 7.6 for 1 h. Sections were then washed with 0.1 M Tris, pH 7.6, and incubated with streptavidin-conjugated horse radish peroxidase (Vectastain ABC kit; Vector Laboratories) diluted in 2% FBS/0.1 M Tris pH 7.6 for 1 h. Sections were washed with 0.1 M Tris, pH 7.6, and then colorimetric development was completed using 3, 3’diaminobenzidine (DAB kit; KPL). Reactions were stopped by immersing the slides in 0.1 M Tris, pH 7.6, and sections were counterstained with Mayer’s hematoxylin (Sigma Aldrich). Slides were dehydrated with an ascending series of ethanol solutions (70–100%) followed by xylenes, and coverslipped using Cytoseal 60 (Thermo Scientific). A subset of tissues were analyzed by Gallyas silver stain, which was performed as previously described [47].

For immunofluorescence, following incubation with primary antibodies, sections were incubated with secondary antibodies conjugated to Alexa fluor 594 or Alexa fluor 488 (Invitrogen, Eugene, OR) followed by Sudan Black treatment and staining with DAPI (Invitrogen, Eugene, OR). The sections were coverslipped with Fluoromount-G (Southern Biotech, Birmingham, AL) and visualized using an Olympus BX51 microscope mounted with a DP71 Olympus digital camera.

### Assessment of pathology

αS inclusion pathology was observed and qualitatively assessed by two independent observers for relative pathology burden and distribution. Astrogliosis and microgliosis were similarly assessed using GFAP and CD11B reactivity, respectively. Mouse pathology maps were completed independently, confirmed, and averaged together. Quantification of αS pathology burden was performed in the pons of M83^+/−^ mice that developed pathology by MSA lysate injection, A53T human αS fibril injection, and WT human αS fibril injection, utilizing Aperio ImageScope (Aperio, Leica Biosystems, IL).

## Results

**Fig. 1:**
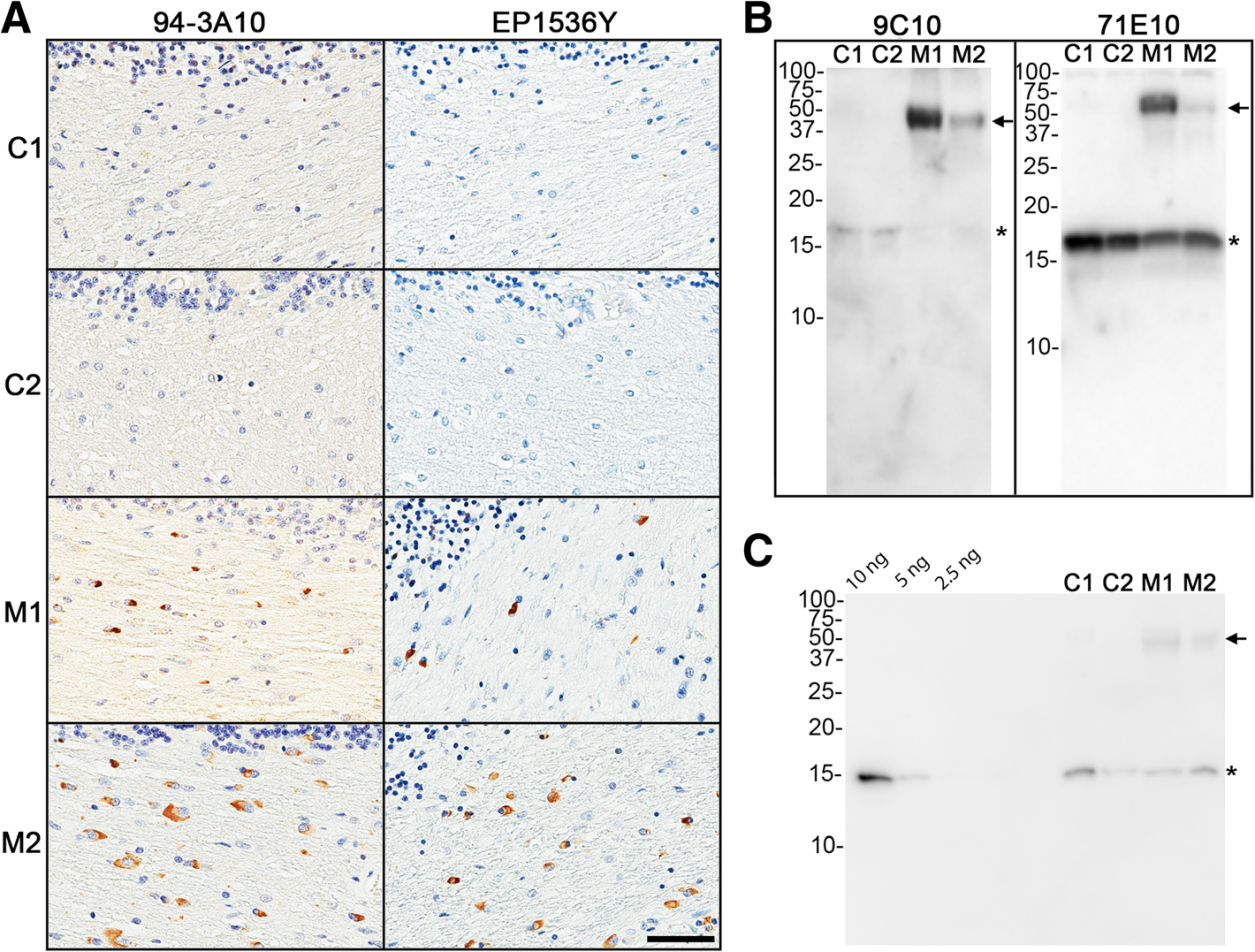
Immunohistochemical and biochemical determination of aggregated αS inclusion pathology in the white matter cerebellum of MSA and control patients used to generate lysates as inoculum. The cerebellum of control (C1 and C2) and MSA (M1 and M2) cases utilized for lysate injections were screened for the absence or presence, respectively, of aggregated pathological αS by immunohistochemistry and western blot analysis. **a** Immunohistochemistry was performed with anti-αS antibody 94-3A10 and anti-pSer129 αS antibody EP1536Y. Scale bar = 50 μm. **b** Immunoblotting was performed with anti-αS antibodies 9C10 and 71E10, where 9C10, preferentially reacts with aggregated αS. **c** Immunoblotting of known amounts (10, 5 and 2.5 ng) of recombinant human αS protein to determine the relative amount of αS within experimental lysates. 2 μL of each sample, as identified above the lanes, was loaded and probed with human αS specific antibody 33A-3F3. For the western blots, the mobility of molecular mass markers are indicted on the left. The band corresponding to monomeric αS is indicated by an asterisks and arrows indicate higher molecular, aggregated αS species. For quantification this analysis was repeated

**Fig. 2:**
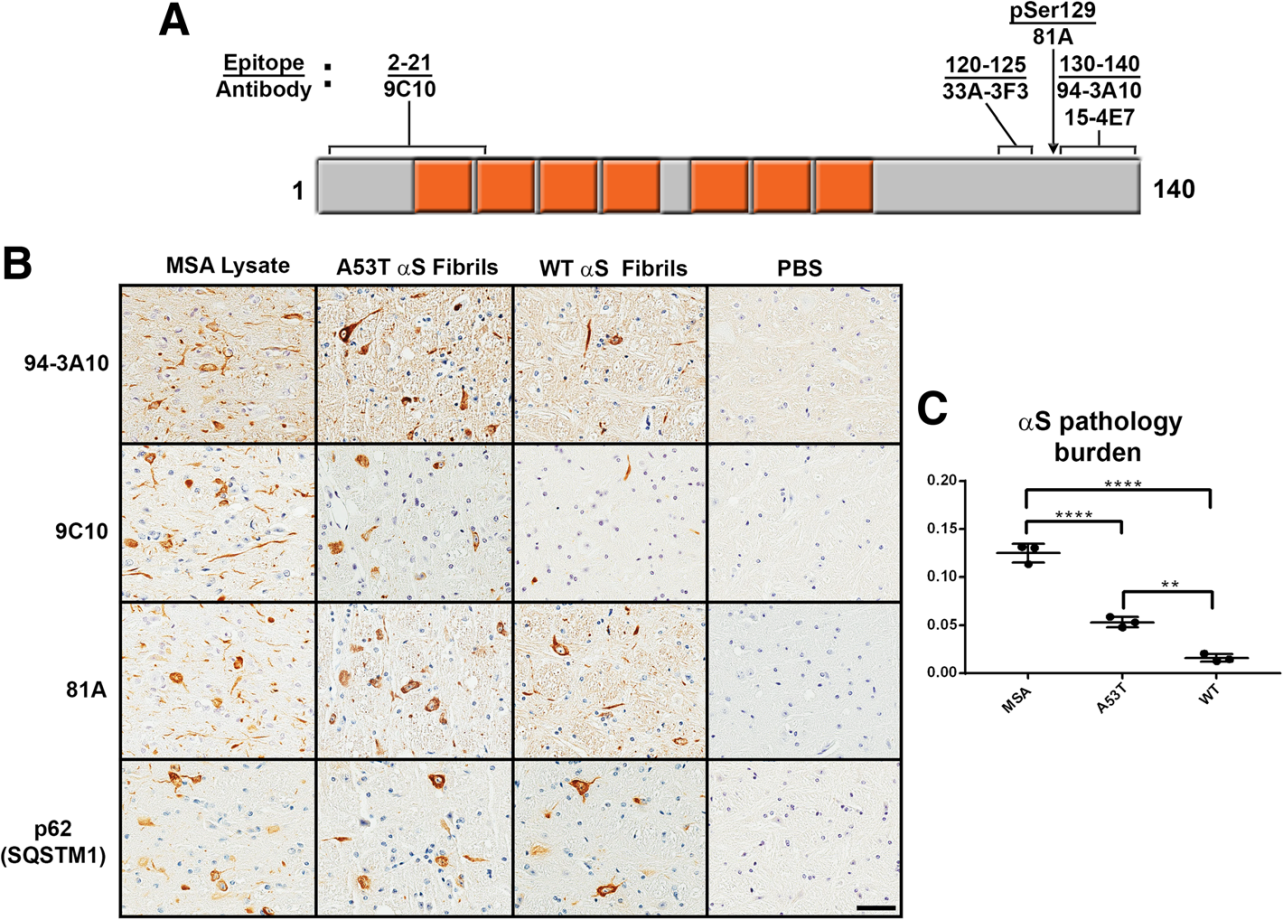
Representative immunohistochemistry of induced αS pathology in M83^+/−^ mice neonatally injected at P0 with PBS, WT human αS fibrils, A53T human αS fibrils, or MSA brain lysates, and quantification of αS pathology. **a** Diagram of the αS protein with the seven imperfect amino acid repeats spanning the N-terminus and hydrophobic middle region in orange. Identified above are the αS antibodies utilized for assessment of αS pathology induction and their respective binding epitopes. **b** M83^+/−^ mice were injected at P0 with as described in “Material and Methods” and aged. Images showing αS inclusion pathology stained with anti-αS antibody 94-3A10 and 9C10 or pSer129 αS antibody 81A in the pons. Sections were also stained with an antibody to p62, a general marker in inclusion formation. **c** Quantification of αS pathology burden in M83^+/−^ mice induced with either MSA lysate, A53T αS fibrils, and WT αS fibrils, performed utilizing antibody 94-3A10 for pathology visualization. ** = *p*-value of <.01. **** = *p*-value of <.0001. Scale bar = 50 μm

**Fig. 3:**
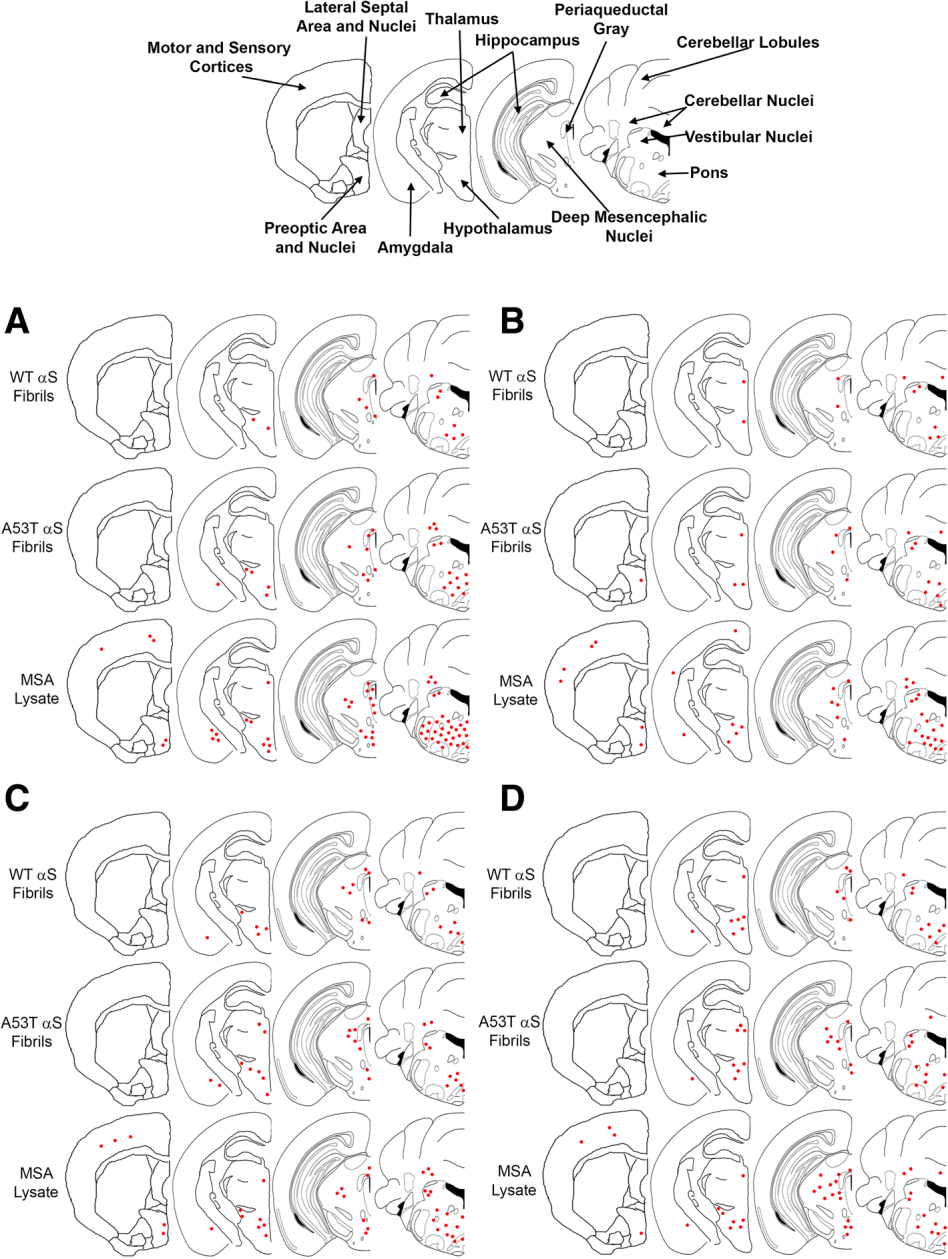
Distribution maps of αS inclusion pathology in P0 injected M83^+/−^ mice. αS pathology distribution in M83^**+/−**^ mice bilaterally injected with 2 μL of either human MSA patient brain lysate (10% w/v), or preformed fibrils comprised of A53T human αS (5 mg/ml) or WT human αS fibrils (5 mg/ml) as stained with antibodies 94-3A10 (**a**), 9C10 (**b**), 81A (**c**) or, anti-62 (**d**). Areas of interest across these studies are labelled in the mouse brain schematic above

Induction of CNS αS inclusion pathology following the direct neonatal mouse brain injection of preformed αS fibrils in nTg and M20^+/−^ mice was previously evaluated by our laboratory for its potential as a high throughput screening method for study of αS pathogenesis using fibrils comprised of recombinant in vitro polymerized human αS [37]. We had shown that M20^+/−^ mice were far more susceptible than nTg mice. In our initial study design, we first expanded this approach to include similar inoculation in the M83^+/−^ mice, as this model is now widely used for assessment of induced αS pathology and transmission [1, 3, 15, 25, 30, 33, 39, 49], and modified the injection location to the lateral ventricles as this would allow for greater spread of the initial seed and therefore greater induction. MSA brain lysates have been reported by several groups to display potent prion-like seeding activity of αS inclusion pathology [2, 28, 30, 43, 49, 54–57]. Using cerebellum tissue from two patients with MSA and similar control tissue from two clinically normal, elder individuals (Table 1), we first confirmed the presence of αS pathology in the MSA cases by immunohistochemistry and western blot analysis (Fig. 1), and generated tissue homogenates for injection, along with PBS injections as a control. Further, we show that the cerebellar lysates used for P0 inoculations contained 5–10 ng of αS (Fig. 1c). Paresis and paralysis are overt motor phenotypes associated with the development of αS pathology in the M83 model and were expected to be prevalent given the previously seen robust nature of the mouse model. Adult M83^+/−^ mice injected with preformed fibrils typically develop robust CNS αS pathology often accompanied by motor phenotypes by 120 days post injection [32, 33, 39]. In our MSA injected cohorts of neonatal M83 mice, 2 of the mice injected with MSA lysate from MSA case 1 began displaying the characteristic hind limb paresis at 5 months of age, and we elected to set ~ 5 months of age as the endpoint for all mice of all genotypes for analysis. None of the other cohorts of mice showed obvious motor abnormalities by 5 months of age. Injection of PBS or brain lysates from control patients did not induce αS pathology in nTg, M20^+/−^ or M83^+/−^ mice (Tables 2 and 3) (see Fig. 2 for example of PBS control injection). At 5 months of age, 25–50% of the M83^+/−^ mice injected with brain lysates from MSA patients M1 and M2 developed αS pathology, while none of the nTg or M20^+/−^ mice showed αS pathology (Table 2). As immunohistochemical analysis revealed the induction of αS pathology only in the M83^+/−^ mice, we utilized an additional cohort of M83^+/−^ mice injected at neonatal day 0 with preformed WT or A53T αS fibrils that were aged to 4 months old, the equivalent of 120 days post injection used in previous injections of adult mice [32, 33, 39], for comparative evaluation of neuropathological features. As expected, the neonatal injection of human WT or A53T αS fibrils in M83^+/−^ mice resulted in the robust CNS induction of αS inclusion pathology (Figs. 2 and 3, Additional file 2: Figure S2, Table 2).

**Table 3:** Summary of αS pathology, microgliosis, and astrogliosis in each mouse line assessed for pathology induction with respective inoculums

	Type of Inoculum	αS Pathology	Microgliosis	Astrogliosis
nTg	PBS	–	+/−	+/−
nTg	Control Lysate	–	+/−	+/−
nTg	MSA Lysate	–	+/−	+/−
M20^+/−^	PBS	–	+/−	+/−
M20^+/−^	Control Lysate	–	+/−	+/−
M20^+/−^	MSA Lysate	–	+/−	+/−
M83^+/−^	PBS	–	+/−	+/−
M83^+/−^	Control Lysate	–	+/−	+/−
M83^+/−^	MSA Lysate	+++	++	+++
M83^+/−^	WT αS Fibrils	+	+	++
M83^+/−^	A53T αS Fibrils	++	+	++

For each mouse line, qualitative neuropathological grading of pathological inclusion burden and immunological response induced by each inoculum was graded on the following scale: (+/−) rare, (+) mild, (++) moderate, and (+++) severe. This grading for M83^+/−^ injected with MSA lysates only include the mice that developed αS inclusion pathology

To assess for inoculum-specific induction of pathology, we utilized several antibodies targeting varied epitopes within αS, in addition to general protein inclusions reactive for p62 (SQSTM1), which colocalizes with ubiquitinated protein aggregates and is thus a marker for proteasome destined targets (Figs. 2 and 3, Additional file 2: Figure S2). We have previously described a panel of αS antibodies that displayed differential reactivity to the αS inclusions present in DLB and MSA [12]. From these studies it was determined that N-terminal antibodies, targeting epitopes within the first 21 residues of the αS protein, preferentially detected neuronal pathology as found in PD and DLB over the GCI pathology found in MSA. Herein, N-terminal antibody 9C10 was utilized to assess if reactivity differences could be identified when comparing MSA lysate injected mice to those injected with preformed αS fibrils. No obvious differences in immunoreactivity between the different injected groups was observed (Figs. 2 and 3, Additional file 2: Figure S2). Of the M83 mice that developed αS pathology, several brain regions were found to be commonly affected, regardless of which inoculum was used for injection. These include the brain stem, vestibular nuclei, cerebellar nuclei, the pons and cerebellar peduncles, periaqueductal grey, deep mesencephalic nuclei, rostral linear nuclei of raphe, superior and inferior colliculi, zona incerta, hypothalamus, thalamus, and amygdala. Sites of αS pathology induction specific for the MSA lysate injected groups were the motor and sensory cortices, which was also seen with general inclusion marker p62 (Fig. 3, Additional file 2: Figure S2). Interestingly, the preoptic area and nuclei displayed an inoculum dependent induction of αS pathology. Using antibodies 9C10 or 33A-3F3 (Fig. 3, Additional file 2: Figure S2), these areas developed immunoreactive pathology when injected with MSA lysate or A53T αS fibrils, but not with WT αS fibrils. Overall burden of αS pathology was greatest for the MSA lysate injected group, followed by mice injected with A53T αS fibrils, with the WT αS fibril injected group possessing the least pathology (Figs. 2b, c and 3). However, the morphology of the pathological inclusions did not differ across groups, as all could be found to possess dense, largely circular, inclusions, with diffuse granular perikaryal aggregates, and pathology within neurites that showed no distinctions across our αS antibody panel (Fig. 2).

**Fig. 4:**
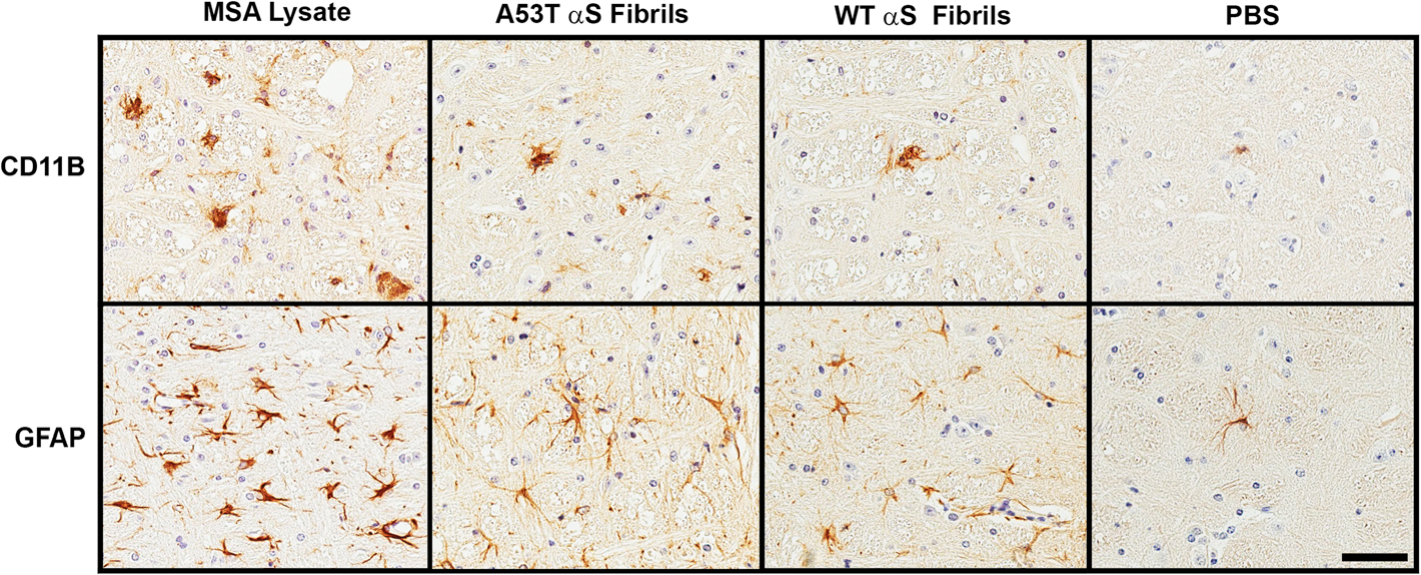
Representative immunohistochemistry of microglia and astrocytes in M83^+/−^ mice neonatally injected at P0 with PBS, WT human αS fibrils, A53T human αS fibrils or MSA brain lysates. M83^+/−^ mice were injected at P0 as described in “Material and Methods” and aged. Images showing microgliosis with an anti-CD11B antibody and astrogliosis with an anti-GFAP antibody in the pons of mice with induced αS pathology. Scale bar = 50 μm

**Fig. 5:**
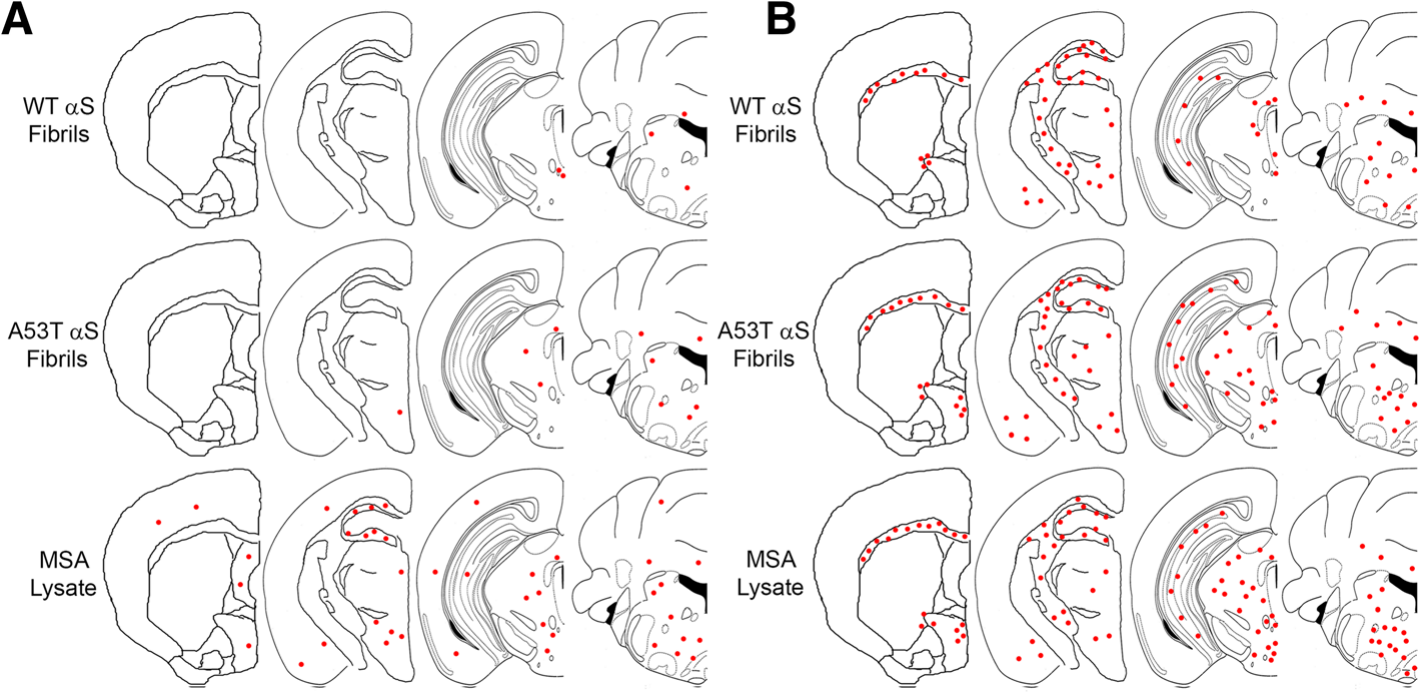
Distribution maps of microglia and astrocytes with respect to each experimental inoculum. Microgliosis (**a**) and astrogliosis (**b**) distribution maps as assessed with antibodies CD11B and GFAP, respectively

The overall location of reactive astrocytosis and microgliosis in M83 mice injected with MSA lysates or preformed fibrils were, with a few exceptions, similar. CD11B immunoreactive microglia and GFAP immunoreactive astrocytes were detected in multiple brain regions in all mice (Figs. 4 and 5). The overall morphology of reactive microglia was similar across the different inoculum used, but we observed that reactive astrocytes in MSA injected mice showed more intense reactivity and appeared more compact (example of observed pathology in the pons in Fig. 4). Reactive astrocyte distribution patterns were consistent across the experimental M83^+/−^ cohorts that developed αS pathology with the exception of the preoptic area in the WT αS fibril injected mice, which did not possess any astrogliosis (Fig. 5b). Microgliosis involvement was considerably more conspicuous in the MSA lysate injected group compared to both WT and A53T αS fibril injected groups (Fig. 5a). Microgliosis found specifically in the MSA injected group could be identified in the motor and somatosensory cortices, the preoptic and lateral septal area and nuclei, the amygdala, hippocampus, thalamus, and cerebellar lobules, demonstrating an immune response unique to MSA white matter lysate. Involvement of the deep mesencephalic nuclei and hypothalamus could not be appreciated in the WT αS fibril injected group, but these structures were affected in the A53T αS fibril injected group. Remarkably, this glial immune cell induction did not always align with the distribution of αS aggregates. For example, the lateral septal nuclei and hippocampus was free of αS inclusions in every mouse observed but microgliosis still occurred in this area for the MSA lysate group. Additionally, αS inclusions could be observed in the hypothalamus and deep mesencephalic nuclei of the WT αS fibril injected group, but no microgliosis was identified. These results would suggest differential induction of the immune system by the components of each inoculum (Fig. 4).

**Fig. 6:**
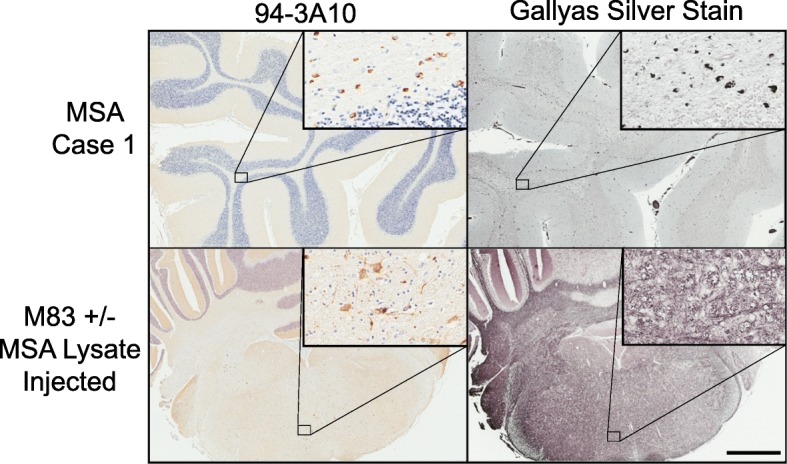
αS pathology induced in M83^+/−^ with MSA lysates is not Gallyas argyrophilic. Representative αS pathology stained by immunohistochemistry with antibody 94-3A10 in the cerebellum white matter of an MSA patient and the pons of an M83^+/−^ mouse following injection with MSA lysate at P0. Gallyas silver staining of adjacent tissue section in the left panels. Scale bar = 2 mm, 50 μm for insets

**Fig. 7:**
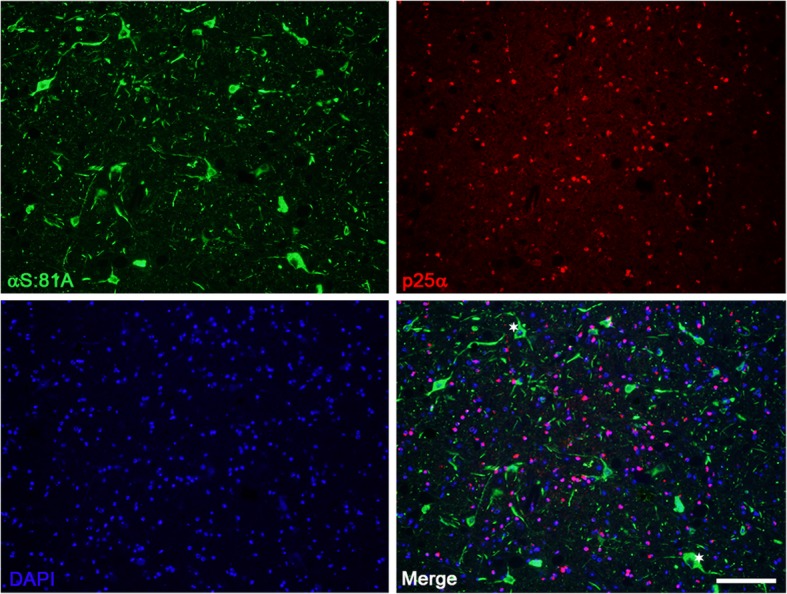
Immunofluorescence analysis demonstrating the paucity of αS pathology within oligodendrocytes of M83^+/−^ mice treated with MSA lysates. M83^+/−^ mice that developed αS pathology following injection with MSA derived cerebellar lysates were evaluated through double immunofluorescence for the cell type within which pathology formed. αS pathology identified with antibody 81A was not seen to co-localize with oligodendroglial marker p25α. Asterisks indicate αS pathology in neuronal bodies. Scale bar = 100 μm

Gallyas silver stain reactivity is a hallmark of GCI pathology in MSA and unlike other silver stains is the only modified procedure that does not detect Lewy bodies [45, 47] (Fig. 6). To further assess MSA-strain like specific induction of αS pathology by the MSA lysates, we performed Gallyas silver staining of the induced αS in M83^+/−^ mice. While the induction of αS pathology in the M83 model by MSA lysate was clearly discernable utilizing αS antibodies, these inclusions were not Gallyas argyrophilic (Fig. 6). For confirmation that the pathology that was induced in the M83 model did not occur within oligodendrocytes, we utilized oligodendrocyte specific antibody p25α [7, 24], and double immunofluorescence with αS antibody 81A (Fig. 7). Aggregated αS observed with antibody 81A could only be found in association with cells that did not possess p25α reactivity, and morphologically appeared as neuronal cell bodies and neurites.

## Discussion

In the present study, we have assessed whether neonatal injection of human MSA brain lysates can induce αS pathology in nTg mice and transgenic mice expressing WT or A53T human αS and if this induction could recapitulate neuropathological features of MSA. Similar to our neonatal seeding studies here, other studies of brain injection using various forms of brain lysates derived from MSA patients into adult M83 mice that express A53T human αS also demonstrated that these mice are permissive to prion-like infection by MSA brain lysates (Table 4) [28, 30, 41, 43, 49, 54–56]. However, the CNS αS inclusion pathology induced from direct brain injection of MSA lysates into M83 mice is consistently typical of the inherent neuroanatomical distribution propensity of αS pathology in these mice [1, 15, 28, 30, 39, 41, 43, 49, 54–56]. These previous studies used varied preparations of MSA brain lysates such as total brain lysates or detergent insoluble fractions, which were both potent inducers of pathology in M83 mice (Table 4). These results on the prion-like seeding activities of various MSA extracts are consistent with a recent study using aggregated αS reporter cells showing that both soluble and insoluble MSA brain extracts have potent seeding activities [57]. Moreover, the MSA lysate inoculations into various peripheral sites induce similar types of CNS αS inclusion pathology in M83 mice (Table 4) [54]. This induced αS inclusion pathology in M83 mice is also associated with motor impairments leading to paralysis consistent with the neuroanatomical distribution of αS pathology [1, 15, 30, 39, 41, 49, 54, 56].

**Table 4:** Summary of investigations utilizing MSA derived brain lysate for in vivo αS pathology induction

Mouse Model	Inoculum Source	αS Preparation	Site of Inoculation	Pathology Induction	References
nTg	Putamen	sarkosyl insoluble	intracerebral	+	Tarutani A et al. [43]
M83^+/−^	Basal ganglia	whole lysate	intracerebral	+ M83 type	Watts JC, et al. [49]
nTg	Basal ganglia	whole lysate	intracerebral	–	Prusiner SB, et al. [30]
Human WT αS/KO^a^	Basal ganglia	whole lysate	intracerebral	–	Prusiner SB, et al. [30]
M83^+/−^	Basal ganglia	whole lysate	intracerebral	+ M83 type	Prusiner SB, et al. [30]
M83^+/−^	Substantia nigra	whole lysate	intracerebral	+ M83 type	Woerman A, et al. [56]
M83^+/−^	Substantia nigra	sarkosyl insoluble/phosphotungstic precipitation	intracerebral	+ M83 type	Woerman A, et al. [56]
M83^+/−^	Substantia nigra or basal ganglia	whole lysate	intraperitoneal	+ M83 type	Woerman A, et al. [54]
M83^+/−^	Substantia nigra or basal ganglia	whole lysate	intramuscular	+ M83 type	Woerman A, et al. [54]
M83^+/−^	Substantia nigra or basal ganglia	whole lysate	tongue	+ M83 type	Woerman A, et al. [54]
M83^+/+^	Substantia nigra	whole lysate	intracerebral	+ M83 type	Sargent D, et al. [41]
KOM2	Unknown brain region	sarkosyl insoluble	intracerebral	+	Peng C, et al. [28]
nTg	Unknown brain region	sarkosyl insoluble	intracerebral	+	Peng C, et al. [28]
M83^+/−^	Substantia nigra or basal ganglia	whole lysate	intracerebral	+ M83 type	Woerman A, et al. [55]
Human WT αS/KO^a^	Substantia nigra or basal ganglia	whole lysate	intracerebral	–	Woerman A, et al. [55]
Human A30P αS/KO^a^	Substantia nigra or basal ganglia	whole lysate	intracerebral	–	Woerman A, et al. [55]
Human A53T αS/KO^a^	Substantia nigra or basal ganglia	whole lysate	intracerebral	+	Woerman A, et al. [55]

Indicated are the human brain regions that was used to generate the MSA lysates containing aggregated αS, the type of lysate preparation, the site of inoculation and the mouse lines used experimentally. Induction of CNS αS inclusion pathology is indicated by a +, with distribution typical of M83 mice indicated as + M83 type. KOM2 are transgenic mice expressing human WT αS specifically in oligodendrocytes [58] and crossed onto a murine αS null background [28]. ^a^Indicates that these transgenic mice are homozygous for the respective form of human αS expressed from a P1 artificial chromosome and are on a murine αS null background [23]

The neonatal injection of in vitro preformed αS fibrils as seeds in M83 mice also induced a similar distribution of αS inclusion pathology. Previous studies of neonatal brain injection of in vitro preformed αS fibrils into nTg and M20^+/−^ mice also revealed induced CNS αS inclusion pathology, albeit that was much more limited in nTg mice [37]. In the current study, within the study design, we were not able to induce CNS αS pathology in nTg or M20^+/−^ mice using MSA brain lysates. The paucity of pathology by MSA lysate neonatally injected in nTg mice is less surprising as in both neonatal and adult brain injection experimental paradigms nTg mice are less susceptible to induction of αS pathology from αS seeding [37, 40, 42]. The lack of induction in the M20 cohort is somewhat more perplexing, as most patients suffering from MSA do not possess αS related mutations, and consequently develop this α-synucleinopathy with exclusively WT αS protein. Both the M20^+/−^ and M83^+/−^ express their respective transgenes at levels 4 to 5 fold higher within the brain [15], which should theoretically be sufficient for creating a permissive environment for αS deposition, as just a single duplication of the *SNCA* locus is sufficient to initiate synucleinopathy disease within humans [9]. Since we have previously shown that induction of αS pathology in M20^+/−^ mice is achievable through preformed fibril injections [37] it is likely that a necessary threshold must be overcome, and that unpurified MSA cerebellar lysate is insufficient in this regard. The M83 mice, on the other hand, possess an autosomal dominantly inherited familial mutation in their αS transgene that was originally described in Italian family that resulted in a much earlier age of disease onset [29], and likely due to its increased aggregation propensity [10] is a direct causal factor in the disease pathogenesis. This renders the insult necessary to overcome this threshold far easier in this model and results in disease that is primarily a result of A53T αS intrinsic nature and less a result of the seed.

The paucity of permissive infection from MSA lysates in nTg mice is consistent with a previous study by the Prusiner group using total brain lysates [30]. The induction of αS pathology in nTg mice has only been shown to be accomplished through purification and enrichment of αS in sarkosyl-insoluble fractions [28, 43]. Like the paucity of induced αS inclusion pathology in M20 mice using total MSA brain lysates, the Prusiner group also reported similar negative infectivity following brain injection using other transgenic mice expressing either WT or A30P human αS [30, 55]. However, in mice expressing human αS with the A53T mutation on a P1 artificial chromosome resulting in a widespread CNS transgene expression, potent induction of αS pathology was obtained with brain MSA lysate inoculation [55]. All these mice express similar levels of human αS and are all on a murine αS null background further supporting the notion that the A53T mutant greatly exacerbates MSA prion-like infectivity [55]. However, the distribution of αS pathology was distinct from that in M83 mice as the induced αS pathology in these A53T αS transgenic mice was predominantly hippocampal and limbic [55]. Nevertheless, similar to studies using nTg [28, 43] and M83 mice [28, 30, 49, 54, 56], induced αS pathology was not of the GCI type, as it was primarily present in neurons.

The only report of predominant induction of αS oligodendrocyte pathology using MSA lysate used αS transgenic mice that selectively expressed human αS in oligodendrocytes and that are on an αS murine null background [28]. Additional cell culture studies demonstrated that MSA derived αS seeds do not show any cell-type preference and that induction of αS aggregation is dictated by the cell types that express αS [28]. Some studies suggest that MSA derived αS has substantially greater potency in seeding activity of αS inclusion formation compared to in vitro preformed αS fibrils, perhaps characteristic of a unique conformer strain [28], but these differences in MSA derived αS was not observed by others [43]. Here we show that the quantity of αS, both monomeric and aggregated forms, in our human cases used to generate inoculum was less than 10 ng, far less than the  10 μg of recombinant αS fibrils used for comparison, indicating that the components of MSA diseased brain lysate contains potent inducers of pathology. An important step in future studies should utilize immunodepletion of αS to determine whether additional factors are responsible for the observable greater induction of pathology, such as those brought about by inflammatory changes.

The previous inoculation studies had utilized MSA lysate inoculum that was retrieved from basal ganglia structures which are known to contain neuronal αS pathology in addition to the characteristic GCIs. In this study, we used cerebellar white matter tissue as our primary inoculum as the MSA pathology in this CNS structure is known to be predominantly GCI based, with little to no accumulation of αS aggregates within neuronal cells, in the hopes that the GCI specific strain induction of αS pathology could be appreciated by removing the possible confounding variable of off-pathway strains. To assess for evidence of putative strain differences in the induction of αS pathology compared to preformed αS fibrils, regional analysis of induced αS pathology was performed with several αS antibodies. Some subtle differences could be observed, such as the more profound involvement of the motor and somatosensory cortices in the MSA lysate injected group. Additionally, the relative activation of astrocytes was considerably greater in the MSA lysate injected mice that developed pathology, along with a greater degree of microgliosis, further supporting inoculum dependent reactions and suggesting a role in the pathogenesis of αS. When comparing relative burden across the M83 injected groups we must consider the age discrepancy between the cohorts. The MSA lysate injected group was allowed to age for an additional month, which is likely a contributing factor in the amount of protein aggregate deposition and the immunological response. Nevertheless, the involvement of specific CNS structures suggests differential responses to each inoculum, or strain-like effects. However, comparative analysis of inclusion morphology did not differ across groups. GCIs are characteristically significantly more Gallyas silver stain positive than neuronal αS pathological inclusions, likely due to structural differences in the aggregated αS in both types of inclusions, [45, 47]. As such, we also investigated if the induced αS pathology using MSA lysates would be more reactive to Gallyas silver staining if induced conformations were maintained, but this outcome was not observed. Furthermore, a lack of colocalization of αS pathology with an oligodendrocyte marker confirms the primarily neuronal tropism of αS pathology within the M83 mouse model.

## Conclusions

These data indicate that MSA brain lysates contain sufficient seeding activity to induce αS inclusion pathology following neonatal injection in M83^+/−^ mice, possibly involving several mechanisms beside conformational templating such as disruption of normal protein homeostasis and neuroinflammatory changes. The intrinsic properties of αS seeds present in MSA or obtained through in vitro fibrillization of recombinant protein, as revealed by the type and distribution of αS pathology, are dominated by the intrinsic transgenic A53T αS strain present in the M83 model. Consequently, these prion-like transmission studies have not yet explained why in MSA αS pathology predominantly accumulates in oligodendrocytes as MSA derived αS does not appear to have the ability to induce strain-like cell-specific αS aggregation. It is therefore likely that in α-synucleinopathy diseases where mutations in *SNCA* gene are not a contributing factor, αS does not represent a typical  prion, where the protein alone is capable of propagating the disease as is the case with the prion protein in diseases like kuru and Creutzfeldt-Jakob disease, but requires a permissive environment or additional disruption of cellular health.

## Additional files

Additional file 1: **Figure S1.** Widespread brain expression of human αS in M20^+/−^and M83^+/−^ mice. Immunohistochemistry showing the widespread and uniform brain expression of human αS in M20^+/−^ and M83^+/−^ mice utilizing anti-human αS specific antibody 33A-3F3 and the paucity of staining in brain section from an nTg mouse. (PDF 1875 kb)

Additional file 2: **Figure S2.** Additional distribution maps αS inclusion pathology in P0 injected M83^+/−^ mice. αS pathology distribution in M83^+/−^ mice bilaterally injected with 2 μL of either human MSA patient brain lysate (10% w/v), or preformed fibrils comprised of A53T human αS (5 mg/ml) or WT human αS fibrils (5 mg/ml) as stained with antibodies 15-4E7 (A) and 33A-3F3 (B). (PDF 2660 kb)

## Abbreviations

CNS: Central nervous system
DLB: Dementia with Lewy bodies
GCIs: Glial cytoplasmic inclusions
GFAP: Glial fibrillary acid protein
MSA: Multiple system atrophy
nTg: non-transgenic
PBS: Phosphate buffered saline
PD: Parkinson’s disease
SNCA: α-synuclein gene
WT: Wild type
αS: α-synuclein
