# Supplementary figures and images for: Comparative analyses of the in vivo induction and transmission of α-synuclein pathology in transgenic mice by MSA brain lysate and recombinant α-synuclein fibrils
Source: Acta Neuropathol Commun. 2019 May 20;7:80. doi: 10.1186/s40478-019-0733-3 (PMC6526622; doi:10.1186/s40478-019-0733-3)

**nTg**

**M20<sup>+/-</sup>**

**M83<sup>+/-</sup>**

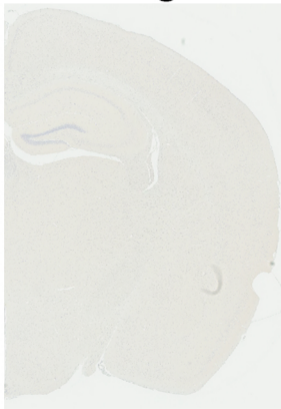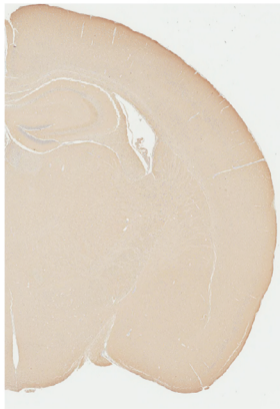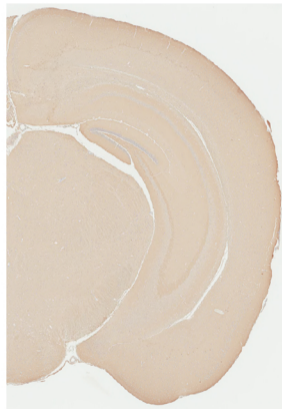

**33A-3F3**

**Supplemental Figure 1**

Supplement: Supplementary file 1 — Figure S1. Widespread brain expression of human αS in M20+/−and M83+/− mice. Immunohistochemistry showing the widespread and uniform brain expression of human αS in M20+/− and M83+/− mice utilizing anti-human αS specific antibody 33A-3F3 and the paucity of staining in brain section from an nTg mouse. (PDF 1875 kb) [file 40478_2019_733_MOESM1_ESM.pdf]
