# Supplementary material for: Comparative analyses of the in vivo induction and transmission of α-synuclein pathology in transgenic mice by MSA brain lysate and recombinant α-synuclein fibrils
Source: Acta Neuropathol Commun. 2019 May 20;7:80. doi: 10.1186/s40478-019-0733-3 (PMC6526622; doi:10.1186/s40478-019-0733-3)
Supplement: Supplementary file 2 — Figure S2. Additional distribution maps αS inclusion pathology in P0 injected M83+/− mice. αS pathology distribution in M83+/− mice bilaterally injected with 2 μL of either human MSA patient brain lysate (10% w/v), or preformed fibrils comprised of A53T human αS (5 mg/ml) or WT human αS fibrils (5 mg/ml) as stained with antibodies 15-4E7 (A) and 33A-3F3 (B). (PDF 2660 kb) [file 40478_2019_733_MOESM2_ESM.pdf]

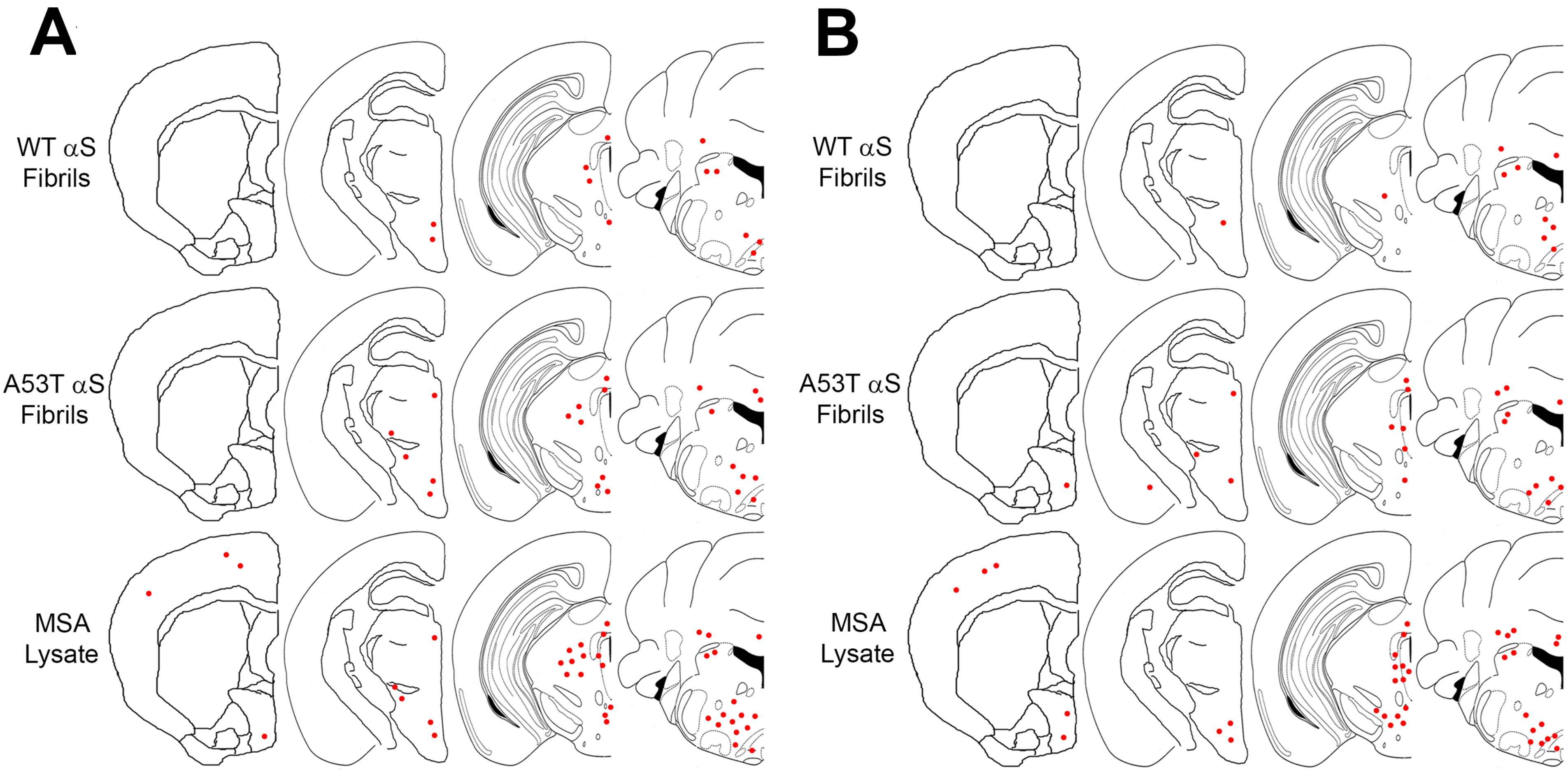

Supplemental Figure 2
